# COVID-19 in Children With Liver Disease

**DOI:** 10.3389/fped.2021.616381

**Published:** 2021-03-11

**Authors:** Angelo Di Giorgio, Steffen Hartleif, Suzan Warner, Deirdre Kelly

**Affiliations:** ^1^Paediatric Liver, Gastroenterology and Transplantation, Hospital Papa Giovanni XXIII, Bergamo, Italy; ^2^Paediatric Gastroenterology and Hepatology, University Children's Hospital, University of Tübingen, Tübingen, Germany; ^3^Institute of Immunology and Immunotherapy, University of Birmingham, Birmingham, United Kingdom; ^4^The Liver Unit, Birmingham Women's and Children's Hospital, University of Birmingham, Birmingham, United Kingdom

**Keywords:** COVID-19, SARS-CoV-2, children, liver disease, liver transplantation

## Abstract

**Background:** The global pandemic caused by novel Coronavirus SARS-CoV-2 disease (COVID-19) is a major threat to the general population and for patients with pre-existing chronic conditions. We report data concerning SARS-CoV-2 infection in children with chronic liver disease (CLD).

**Methods:** A literature review using the online database PubMed was performed to summarize available findings on the association between pre-existing liver disease and COVID-19 infection in children.

**Results:** Children with COVID-19 have preserved effector and immunosuppressive components resulting in a milder disease compared to adults. The most common hepatic manifestation is an elevation of hepatic transaminases. Liver damage may be directly caused by viral infection of liver cells, by medications or by the chronic hypoxia seen in COVID-19 patients. A multicenter study reported that the majority of children with a CLD remained healthy during the outbreak. Similarly, studies reported that children on immunosuppressive treatment, including patients with autoimmune liver disease (AILD) and liver transplantation (LT), maintained good health during the outbreak without experiencing major complications even if infected with COVID-19.

**Conclusion:** COVID-19-related liver injury presents with a mild elevation of transaminases, although its clinical significance is unclear. Children with CLD, including those with AILD and post-LT, do not have an increased risk for severe disease course of SARS-CoV-2 infection with little or no liver dysfunction. These data highlight the necessity to ensure normal standards of care while adhering to national Covid-19 guidelines, and particularly to maintain immunosuppressive medication to prevent relapse or rejection. Further research is required to evaluate the differences in clinical course between immunosuppressed adults and children and in particular whether asymptomatic infection is a concern.

## Introduction

The novel Severe Acute Respiratory Syndrome Coronavirus (SARS-CoV-2) disease (COVID-19) is a newly identified illness characterized by symptoms of viral pneumonia, abnormal coagulation, and potentially long term damage to heart, liver and kidneys (1). The current coronavirus pandemic is a major cause of morbidity and mortality in older adults, particularly in males, those with co-morbidity and from a minority ethnic back ground. Currently, it appears that children have a milder illness with significantly less need for inpatient admission or respiratory support as was demonstrated in previous pandemics including influenza A H1N1 (2). It is possible that differences in gender, population and age response may be due to different strains or mutations of the Coronavirus (3) or to differences in immune reactivity. In addition, children are less likely to have the multiple co-morbidities present in older adults (4). Immunocompromised children, such as children with liver disease are considered to be more at risk of Covid-19 although initial experience in Italy has not demonstrated significant problems as yet.

In this review we summarize the recent studies reporting data on: (a) immune aspects of COVID-19 in children with liver disease; (b) COVID-19-related liver injury in children; (c) and characteristics of COVID-19 infection in children with chronic liver diseases including autoimmune liver disease (AILD) and liver transplantation (LT).

## Methods

A systematic search was performed using online PubMed, Embase, Cochrane Library, and pre-prints platform to identify all relevant studies on children with a CLD and COVID-19; a combination of the following key words was used: “SARS-CoV-2” OR “COVID-19,” AND “children” OR “autoimmune liver disease” OR “hepatic manifestations” OR “chronic liver disease” OR “liver transplantation” in all fields between 2019 and present time.

**Figure 1:**
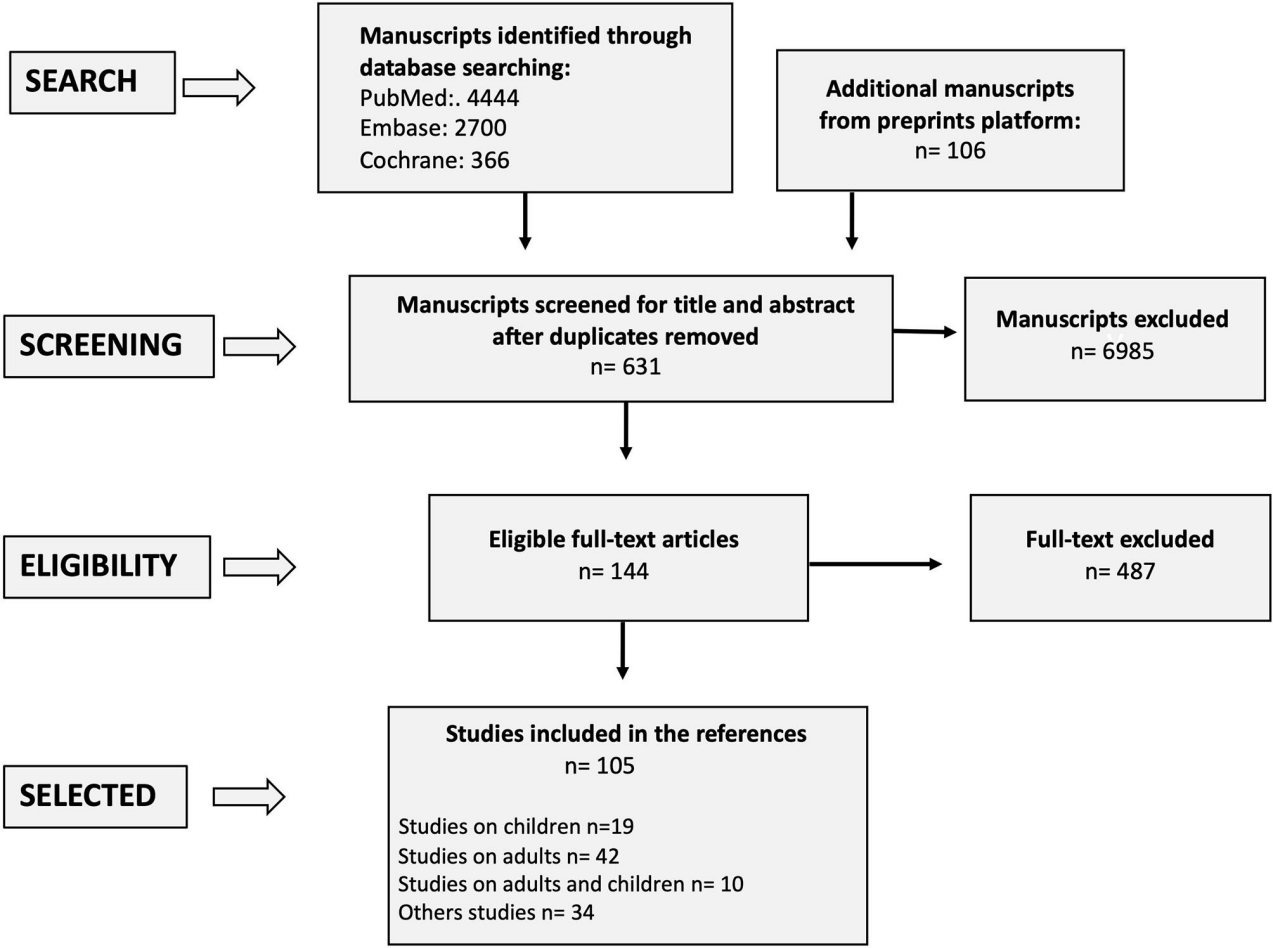
PRISMA flowchart.

In absence of strong evidences from the pediatric literature, studies from adult patients were considered. Following the removal of duplicates or overlapping publication, title and abstract screening were performed by all authors to identify potentially eligible articles. Among these studies, we selected and included in the references the available full-text articles focusing on: (a) immune aspects of COVID-19 in children with CLD; (b) hepatic manifestations in children with COVID-19; (c) COVID-19 in children with CLD, (d) COVID-19 in children with AILD, and (e) COVID-19 in pediatric liver transplant recipients. The selection process is presented in the Figure 1.

## Results

A total of 7,616 records were identified through the comprehensive search, from which 144 full texts were reviewed, and 105 studies were considered suitable for this study and included in the references. Seventy-one of 105 studies were on patients with COVID-19 (*n* = 19 studies on children, *n* = 42 on adults, *n* = 10 on children and adults) and 34/105 included immunological and virological studies on SARS-CoV-2, editorial and review articles. The other studies (*n* = 487) were excluded because: (a) they did not report data on children with CLD and COVID-19; (b) the results were overlapping to those already published from other studies.

The selection process is presented in Figure 1. The most relevant studies reporting data on children with a pre-existing liver disease and COVID-19 are presented in Table 1.

**Table 1:** The most relevant reviewed manuscripts on children with CLD and COVID-19.

**References**	**Country**	**Studydesign**	**Total number of patients**	Age (years)^*^	Confirmed COVID^**^	**Outcome** ^**^		
						**Hospitalization**	**ICUadmission**	**Survival**
Singh et al. (5)	United States	R	*n* = 2,780 (*M* = 38%); *n* = 250/2,780 (9%) with CLD; all patients aged >10 years; percentage of children aged 10–18 years: not reported	55.2 (±14.6)	Not reported	Not reported	Not reported	Not reported
Di Giorgio et al. (6)	Italy	R	*n* = 369 children with CLD (*M* = 60%)	11.1 (±7.7)	2	0	0	All survived
Di Giorgio et al. (7)	Italy	R	148 patients; 47/148 (32%, *M* = 40%) children with CLD	12.3 (2.8–17.8)	0	NA	NA	NA
Heinz et al. (8)	United States	C	Case report: onset 4 days post-LDLT (Donor and recipients Sars-CoV-2 positive) → Mild respiratory symptoms → Graft hepatitis	6 month	1	1	1	Survived
Morand et al. (9)	France	C	Case report: 5 month post-LT → Mild respiratory symptoms: tachypnea → Mild Graft hepatitis	4	1	1	0	Survived
Dona et al. (10)	Europe	R	a) Survey by 18 pediatric transplant centers (solid organ and stem cell transplantation) b) Individual follow-up of 14 affected children (including stem cell transplantation and waiting list) and 5 solid organ transplant recipients	a) 0–17 years (no details reported)b) Not reported	a) No absolute numbers reported b) 14 cases, 5 solid organ transplant recipients	a) 10/18 centers reported <10 COVID-19 pediatric cases b) 14 cases, 5 solid organ transplant recipients	a) <5% of affected children b) Not reported, but 2 severe courses	a) All survived b) No details reported
Nicastro et al. (11)	Italy	R	Survey of 155 pLT recipients in the region of Lombardy	14.8 (0.3–17.9)	0 (2 suspected cases with close contact)	0	0	All survived
D'Antiga et al. (12)	Italy	R	Cases series of 3 Sars-CoV-2 positive patients out of a cohort of *n* = 700 pediatric LT recipients	Not reported	3	0	0	All survived

* Mean or median;

** *data on children alone; C, case report; R, retrospective; P, prospective; M, male; NA, not applicable*.

### Immune Aspects of COVID-19 in Children With Liver Disease

There is great anticipation and expectation placed on the much awaited global SARS-CoV-2 vaccination program which, if successful, may bring about social and economic restoration. It is even more important that we understand the pathogenesis of this devastating infection which intriguingly affects adults and the elderly more than children (2–4). There is insufficient data on the virulence and clinical course of the new SARS-CoV-2 variant, which appears to be more infectious and spreading faster among children and adolescents as well as in adults (13, 14).

The pathogenesis and immune aspects of the Coronavirus disease 2019 (COVID-19) are briefly discussed below.

Coronavirus dissemination in humans is via droplets and aerosol spread. The viral capsid spike (S) glycoprotein of SARS-CoV-2 gains entry via angiotensin-converting enzyme II on the surface of type 2 pneumocytes (15, 16). TMPRSS2, transmembrane serine protease 2, helps to prime the spike protein and promotes entry. Once inside the host cell, viral replication takes place, releasing virions which infect other ACE2 expressing cells (15–17).

One function of ACE2 is to convert angiotensin I into angiotensin 1-7. Ang 1-7 is anti-inflammatory and anti-fibrotic, thus protecting against pulmonary capillary leak and inflammation (17, 18). Many studies report children to have higher ACE2 expression than adults, an important factor thought to explain why children are more resilient to COVID-19 (4, 17, 18). Not all studies agree with this finding, as downregulation of ACE2 upon SARS-CoV-2 spike protein binding is described in adult patients with comorbidities such as hypertension and diabetes (17, 19).

There are other differences in the immune response to SAR-CoV2 infection in adults and children, apart from ACE2 expression, particularly.

To begin with, the lymphopenia and high neutrophil to lymphocyte ratio described in adults with severe COVID-19 infection are not observed in children (20, 21). Lymphopenia is found in up to 80% of adults, and affects all lymphocyte subsets including T cells, B cells and natural killer cells (NK), but chiefly affects cellular immunity with T cell suppression (16, 20–22). The suppression is not only in numbers but in expression of cell exhaustion and inhibition markers such as PD-1, LAG-3 and TIM3 (16, 22). Children with COVID-19 appear to have preserved CD8+ cytotoxic responses except in the most severe of cases (4, 19, 21). This may be in part explained by immunosenescence which occurs as part of normal aging and is more prominent in the elderly, causing a latent and suboptimal adaptive response to microbes (4, 19).

A case study report of mild COVID-19 infection in a 5 year old boy with newly diagnosed autoimmune hepatitis (AIH) and a background of well-controlled diabetes mellitus, was interesting. As opposed to inhibitory markers, this patient had a higher percentage of the activation marker HLA-DR+ on CD8 lymphocytes at COVID-19 onset which declined with recovery (23). Azathioprine was stopped but low dose oral prednisolone continued and his liver function remained normal throughout.

Adults with severe and life-threatening COVID-19 also have higher circulating pro-inflammatory cytokines. This cytokine storm is preceded by pulmonary infiltration of monocytes/macrophages and neutrophils. These innate cells secrete powerful inflammatory cytokines including interleukin-1B (IL-1B), IL-6, IL-12, IL-10, IFNg, and TNFa (16, 20, 22, 24). The chemokines CXCL10, CXCL8, and CCL2 are likewise detected in large amounts.

Levels of IL-6, IL-10, and C-reactive protein (CRP) correlate and predict disease severity (20, 22, 24). Lower levels of these cytokines and CRP are found in children with COVID-19. Indeed, a child's innate immune system is still undergoing maturation and thus may not launch as strong a response as adults (4, 19, 22).

Wu et al. studied 157 pediatric COVID-19 cases. In contrast to adults, IL-6, IFNg and TNFa levels were found unchanged although IL-10, an immunosuppressive cytokine was elevated in both adults and children (21, 25, 26).

Pierce et al. in New York City on the other hand, found children with COVID-19 (*n* = 65) had higher IFNg and IL-17, and unchanged IL-6 and TNFa levels compared to adults with COVID-19 (*n* = 60) (27). These children had a shorter hospital stay, were less likely to require mechanical ventilation and had lower mortality (27). Higher IFNg is notable as it is a very important anti-viral cytokine produced by both innate cells, CD4+ T-helper 1 cells and CD8+ cytotoxic T cells (28, 29). IL-17 is a cytokine produced mainly by T-helper 17 cells and functionally has a role in mucosal integrity and airway re-modeling (30, 31).

Firm conclusions cannot be drawn from so few studies but the data support the theory that children have preserved adaptive immunity and do not mount as pronounced an innate response as adults.

Other factors to consider in age related immune differences include the role of cross reactive antibodies and trained immunity.

Cross reactive antibodies from common viral upper respiratory tract infections are hypothesized to provide protection against microbes with similar antigenic epitopes (4, 19). This is especially applicable to the coronaviruses as, of the seven which infect humans, four commonly infect children; NL63, 229E, HKU1, and OC43 (18). A recent pediatric study of 244 children who all tested negative for COVID, found 36 had close COVID-19+ve contacts. Seven of the 36 patients (mean age 5years) were positive for Mycoplasma pneumoniae and another 6/36 were positive for the human metapneumovirus on nasopharyngeal swabs. Studies have further shown children testing negative for SARS-CoV-2 had higher IgG antibody titres against Respiratory syncytial virus (RSV) and/or M. pneumoniae (32). These findings support the theory that common childhood infections may offer cross protection (33).

Cross protection has been classically described with the Bacillus Calmette–Guérin (BCG) vaccination but is speculated to occur with others vaccines (4). The BCG vaccine augments CD4+ T helper 1 immunity and has been demonstrated to provide cross over immunity (34, 35).

Trained immunity is a relatively new concept describing the functional reprogramming of innate immune cells following their epigenetic modification from vaccinations and common childhood infections, enabling memory cell formation which was previously considered unique to the adaptive immune system (4, 19).

Taken together, the evidence suggests adults with COVID-19 may have an activated but ineffective cellular response, combined with an over stimulated innate immune response and uncontrolled pro-inflammatory cytokine production. Children with COVID-19 appear to have preserved effector and immunosuppressive components of the adaptive cellular response and do not exhibit the cytokine storm which is so prominent in adults.

Despite reports of mild illness in children, the Kawasaki like illness, pediatric multi-system inflammatory syndrome (PIMS), aka multi-system inflammatory syndrome (MIS-C), developed in 2.1% of all COVID-19 cases in children [The European Surveillance System (TESSy)] (27). There was a lag time of 4–5weeks after the peak of COVID-19 before PIMS cases were reported. Children with PIMS were older (>5 years), had more cardiac involvement and cytokine storm activation than patients with classical Kawasaki disease (36). Although many children were admitted to Intensive care, mortality was low (27, 36). Of note, children with a genetic under-expression of ACE2, are thought more likely to develop PIMS (20), which strengthens the notion that ACE2 is protective against COVID-19 in children.

In addition, the differences and similarities in comorbidities between children and adults may also be a factor. Obesity is a recognized risk factor in both children and adults with severe COVID-19 (37, 38). Hypertension and diabetes is not as commonly reported in children, but reflects the lower prevalence of these conditions and the metabolic syndrome in children (37–39). Children with asthma, congenital heart disease, inherited metabolic conditions (inborn errors of metabolism) and syndromic/chromosomal disorders are at higher risk of severe COVID-19 (39, 40), as are children from ethnic minority backgrounds, who are more likely to develop severe PIMS-TS/MIS-C post-COVID-19 (41).

Finally, even in adults, the liver is rarely severely affected and this may be because it has a very tolerogenic microenvironment, with an abundance of regulatory cells such as Tregs, Bregs and tolerogenic dendritic cells along with the immunosuppressive cytokines they produce e.g., IL-10, IL-35, and TGFB (42–44). This may underpin its resilience to COVID-19 infection in addition to or despite of the predominance of ACE2 expression found on cholangiocytes and to a lesser extent, hepatocytes (45, 46).

### COVID-19 and Hepatic Manifestations

SARS-CoV-2 may cause a systemic disease with possible involvement of other organs including the liver because of ubiquitous distribution of the main viral entry receptor, namely angiotensin converting enzyme 2 (ACE) (1, 47). We report data on the characteristics of COVID-19-related liver injury and its correlation with clinical outcomes.

COVID-19-related liver injury is defined as any liver damage occurring during disease progression and treatment of COVID-19 in patients with or without pre-existing liver disease (48). In COVID-19 patients the most common hepatic manifestation is represented by elevated hepatic transaminases, both aspartate aminotransferase (AST) and alanine aminotransferase (ALT); mild elevations in gamma-glutamyl transferase (GGT), alkaline phosphatase (ALP) and total bilirubin are also reported, although less frequently. Serum albumin is decreased in severe cases although a significant impairment of liver function as the cause of death in COVID-19 rarely occurs (49–51).

In adults with COVID-19, the incidence of elevated transaminases is reported between 14 and 53% (48). In Guan's study (*n* = 1,099 patients), the elevation of AST and ALT was of 22.2 and 21.3% of cases, respectively; a small proportion of patients (76/722, 10.5%) presented also abnormal bilirubin levels (49).

Few data are available on children. In Qiu's study only 2 out of 36 children (6%) with COVID-19 [mean age 8.3 (±3.5), *M* = 64%] had an elevation of transaminases (52). In another study from China (*n* = 31 children, median age 7.1 years, range 0.6–17), the rate of elevated transaminases was of 22% being the highest value registered of ALT and AST 68 UI/L and 67 UI/L, respectively (53). In both studies no patients developed severe liver impairment. Zhu et al. analyzed the clinical features and outcomes of 10 neonates born to mothers with COVID-19 pneumonia and reported only 2 patients (20%) with abnormal liver function tests (54). Similarly, at the Hospital Papa Giovanni XXIII, Bergamo, Italy, which is one of the largest European centers for pediatric liver transplantation and it was located in the “red zone” for the Italian outbreak, a slight increase of ALT was observed in only 9/33 children (27%, aged 1–17 years) and in 2/9 infants with COVID-19 (22%, all aged <3 months); liver synthetic function was preserved and transaminases normalized spontaneously in all patients without requiring any treatment (personal data, unpublished).

In pediatric COVID-19 reports, increasing levels of serum interleukin (IL)-6 and IL-10 are also associated with greater COVID-19 disease severity, although the levels of these cyto- kines are not significantly different between infected children with and without elevated serum liver enzymes. This suggests that interleukins do not play a key role in COVID-19 related abnormal liver enzymes amongst pediatric patients (55).

Of interest, in Cai's study (*n* = 417 patients with COVID-19; *n* = 20 were children aged <10 years) the authors reported a high proportion of patients with elevated transaminases (318/417, 76.3%) suggesting that the liver damage might be directly caused by viral infection of liver cells (56).

Other studies reported that from 1 to 11% of patients admitted with COVID-19 had chronic liver disease suggesting that abnormal transaminases could also result from pre-existing chronic liver disease (57). These suggests that pediatricians should consider underlying liver disease in children with COVID-19 and abnormal liver enzymes (57).

The association between liver injury and adverse clinical outcomes in COVID-19 patients is unclear. In adult studies, higher levels of transaminases were found in patients with severe COVID-19 compared to milder cases. In Guan's study (*n* = 1,099 patients), elevated levels of AST were observed in 112 (18.2%) patients with mild disease and in 56 patients (39.4%) with severe disease (49). Similarly, in Huang's study the proportion of liver injury in intensive care unit (ICU) patients (61.5%) was higher than in non-ICU patients (25.0%) (1). In a recent meta-analysis on 2,115 patients, the authors found that patients with liver injury had a more severe disease (OR: 2.57, 95% CI 1.25 to 5.26, I2 = 62%, *p* = 0.01) and a higher prevalence of mortality (OR: 1.66, 95% CI: 1.04 to 2.64, I2 = 35%, *p* = 0.03) compared to those without liver injury (58). Contrasting data were reported in other studies, which did not find a significant association between liver injury and mortality, disease progression, ICU admission, or length of hospital stay, so that, to date, there is no strong evidence to support a “causal relationship” between liver damage and severe course of COVID-19; further studies are needed to better define this association (59–61).

The mechanism of liver injury in patients with COVID-19 is also unclear (62). Liver histology in deceased patients with COVID-19 demonstrated that histopathological findings are highly suggestive for marked derangement of intrahepatic blood vessel network secondary to systemic changes induced by virus that could target not only lung parenchyma but also cardiovascular system, coagulation cascade and endothelial layer of blood vessels (63).

Liver damage could be directly be caused by viral infection of liver cells because SARS-CoV-2 utilizes the angiotensin-converting enzyme 2 (ACE2) as docking and entry receptor on host cells. Based on single-cell sequencing and animal model analysis of liver tissue, the specific expression of ACE2 in bile duct epithelial cells was 20 times higher than that in hepatocytes suggesting that SARS-CoV-2 might directly bind to ACE2-positive cholangiocytes to dysregulate liver function (45). In contrast, pathological analysis of liver tissue from a patient who died from COVID-19 did not show the presence of viral inclusions in the liver (63).

Many medications used to treat COVID-19 patients are potentially hepatotoxic. Fan and co-authors found that 48% of 148 COVID-19 patients developed LFTs abnormalities 1 week after admission suggesting a hepatotoxic mechanism of the medications administered (59). In other studies, the antiviral treatments for SARS-CoV-2, lopinavir/ritonavir or Remdesivir were considered the cause of liver injury in patients with COVID-19 (56, 57, 64).

As detailed above, the levels of IL-2-receptor (IL-2R) and IL-6 are significantly increased in the serum of COVID-19 patients and correlate with disease severity (65). Lu et al. proposed that lymphocytopenia and C-reactive protein levels were independently correlated with liver injury in COVID-19 patients suggesting that the main mechanism of liver damage might be an inflammatory cytokine storm (66, 67). The pathogenetic mechanisms of liver damage in either adults or children are not fully understood and the impact of COVID-19- on the clinical outcomes is undefined. Further research is needed to define liver involvement in children with COVID-19.

In conclusion, COVID-19-related liver injury in children consists of a mild elevation of transaminases, although its clinical significance is unclear. As COVID-19 is not always associated with abnormal liver biochemistries, all children with elevated hepatic transaminases should be evaluated for underlying liver disease and/or coexisting infections.

### COVID-19 in Chronic Liver Disease

The novel Coronavirus SARS-CoV-2 disease (COVID-19) pandemic threatens both the general population and patients with pre-existing chronic conditions. However, it is unclear whether patients with a chronic liver disease (CLD) are more susceptible to SARS-CoV-2 infection or are at higher risk of more severe disease (68). In this section we explore the interaction between COVID-19 and CLD, and the impact of COVID-19 on patients with liver chronic conditions with particular attention to the pediatric population. The incidence of CLD in patients with COVID-19 ranges from 0.6 to 37.6%. In the majority of studies, there are few details of the diagnosis or extent of pre-existing liver conditions making it difficult to assess the impact of COVID-19 on patients with different types of liver disease (49, 51). Overall, it is reported that an underlying CLD is associated with a worse outcome of COVID-19 (69, 70). Patients with cirrhosis are known to be at increased risk of decompensation or development of acute-on chronic liver failure if infected with bacterial, fungal or viral infection, and so it is likely that cirrhosis will be a risk factor for severe COVID-19 (68, 71). In Singh's study (*n* = 2,780 patients from United States), patients with pre-existing liver diseases were found to have an increased risk of mortality (RR, 2.8; 95% CI, 1.9–4.0; *p* < 0.001) compared to patients without liver disease. The relative risk was higher in patients with cirrhosis (RR, 4.6; 95% CI, 2.6–8.3; *p* < 0.001). Although, children aged > 10 years were included in this study, no clinical details were reported, so the outcome is not clear (5). In a large study on 745 patients with CLD and COVID-19 from 29 countries (*n* = 386 with cirrhosis and *n* = 359 without, all adults) the mortality was significantly higher among the cirrhotic patients (32 vs. 8% in non-cirrhotic, *p* < 0.001) (72).

Guan's study (*n* = 1,099 patients; 9 aged <14 years) provided data on chronic hepatitis B (CHB) and COVID-19 and indicated that only one adult patient had severe disease suggesting that CHB does not affect the outcome of COVID-19 (49).

In view of the adverse effect of COVID-19 on patients with associated comorbidities, it is possible that patients with non-alcoholic fatty liver disease (NAFLD) or steatohepatitis (NASH) who have associated diabetes, hypertension and obesity, will have a severe course of COVID-19 (73). In Ji's study, adult patients with NAFLD had a higher risk of progression to severe COVID-19 and had a persistently longer viral shedding time (50). Of interest, in Sachdeva' study, the authors found that NAFLD is a predictor of severe COVID-19 regardless the presence of obesity (74).

In Sarin's study (228 adult patients) patients with cirrhosis were at risk of acute decompensation and those with associated comorbidities were more vulnerable and required close monitoring (75). Similar data was reported in Cai's study (*n* = 298 patients; only 20 (6.7%) aged <10 years) and in a recent position paper by the European Association for the Study of the Liver (73, 76). Studies of adult patients with liver cancer have shown that they are at increased risk for severe COVID-19 and have a poorer prognosis because of their systemic immunosuppressive state and treatments, such as chemotherapy or surgery, suggesting that they require more intensive surveillance and early admission in case of COVID-19 co-infection.

It is not clear why there are differential outcomes among some subgroups of patients with CLD. It is possible that the ACE2 receptor, which is expressed in bile duct cells, might favor viral entry and cause the liver damage. Additional mechanisms include drug induced liver injury and the chronic hypoxia seen in COVID-19 (61). There are few data on the health status of children with CLD during the pandemic and no pediatric studies on the interaction between a pre-existing liver disease and COVID-19, and therefore the impact of COVID-19 on clinical outcomes of children with CLD is unknown.

In a recent multicenter study, we performed a phone-based survey using a questionnaire to explore the characteristics of COVID-19 in children with CLD followed up at three hepatology centers in Italy, between January and June 2020. We defined suspected COVID-19 case the presence of at least one of the following two conditions: (1) An episode of acute respiratory tract infection (RTI) (sudden onset of at least one of the following: cough, fever, shortness of breath); (2) A close contact with a confirmed or highly probable case of SARS-CoV-2 infection. We defined confirmed COVID-19 as having a positive nasal-pharyngeal swab (NPS) for SARS-CoV-2 nucleic acid using real-time reverse-transcriptase polymerase-chain-reaction (RT- PCR) assay (77). The observed incidence was weighted to expected cases using previously published models (78).

Three hundred and sixty-nine out of 377 patients responded to the survey (Survey Response Rate = 98%). Mean age was of 11.1 years (±7.7). Diagnoses included both cirrhotic and non-cirrhotic liver diseases. Fifty-six of 369 patients (15%) were suspected COVID-19, 43/56 (77%, mean age 11.3 years, ±8.1, *M* = 49%) had mild respiratory symptoms including fever in 28 patients (65%), cough in 23 (53%), shortness of breath in 4 (10%). Overall, of 30 patients who had a close contact with a highly suspected (*n* = 25) or confirmed COVID-19 case (*n* = 5), 17 (57%) developed respiratory symptoms and 13 (43%) remained asymptomatic. Two patients (0.5%, aged 6 and 18 years, 1 with non-cirrhotic portal hypertension and 1 with biliary atresia) were confirmed COVID-19 cases, both were asymptomatic. Overall, the observed incidence of COVID-19 cases was higher than that estimated in the general pediatric population. Nevertheless, symptoms were mild in all, none required hospitalization and all children (100%) survived, (Table 2). The results from this study demonstrated that the majority of children with CLD (84%) remained healthy during the outbreak. Despite a high incidence of observed suspected cases, the absence of major illness and a favorable outcome, even in confirmed COVID-19 cases, suggest that in children, an underlying liver disease does not represent an additional risk factor for severe disease (6).

**Table 2:** Demography, clinical features, and COVID-19 of children with chronic liver disease (6).

**Number of patients**	369
**Survey response rate°**	98%
**Male (%)**	220 (60%)
**Age at survey, years (mean)**	11.1 (±7.7)
**Chronic liver disease**	
- Biliary Atresia	91 (25%)
- Chronic Viral Hepatitis B or C	79 (22%)
- Vascular disorders	42 (11%)
- Alagille syndrome	29 (8%)
- Metabolic disorders	20 (5%)
- Other conditions	108 (29%)
**Suspected cases of COVID-19, *n* (%)**	56 (15%)
- Mild respiratory symptoms	43
- Asymptomatic	15
- Close contact with a highly suspected or confirmed COVID-19	30
Confirmed cases of COVID-19^*^, ***n* (%)**	2 (0.5%)
**Outcome**	
- Survived	369 (100%)
- Patients requiring hospitalization	None

° It indicates the percentage of patients who responded to the survey; COVID-19, Coronavirus SARS-CoV-2 disease.

* *Both patients had a nasopharyngeal swab positive for SARS-CoV-2, both were asymptomatic*.

**Figure 2:**
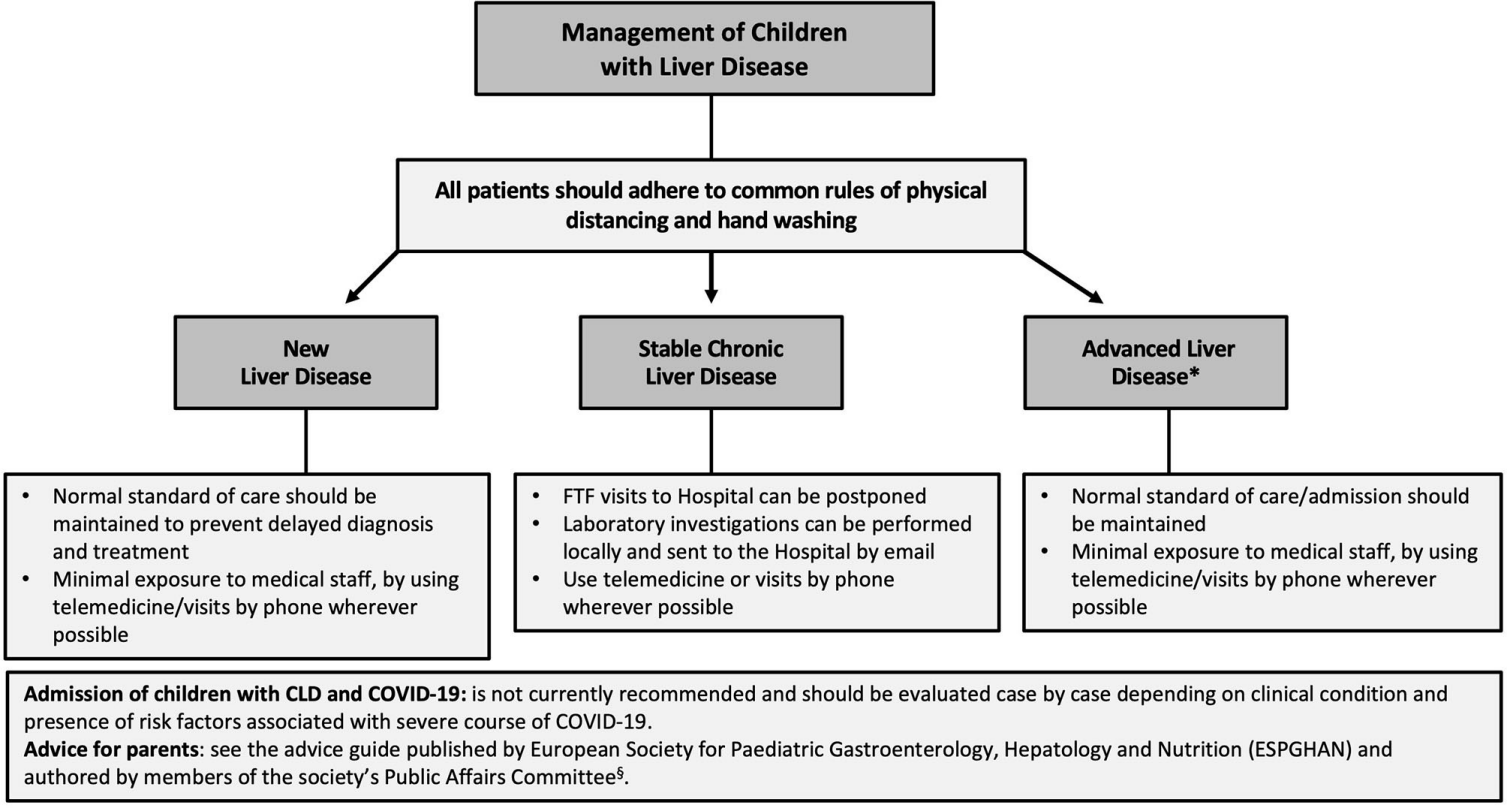
Flow chart for the management of children with chronic liver disease (CLD) during the COVID-19 outbreak. Individual management of these patients strongly depends on the local COVID-19 burden and officially implemented rules and regulations. *It includes children with an impaired liver function (coagulopathy and/or low serum albumin) or complications of portal hypertension (ascites and/or gastro-esophageal varices). FTF, face-to-face.

There are no recommendations for the management of children with CLD during the outbreak, but based on the adult experience, we propose that children with stable CLD could be managed virtually by telemedicine in the short term, but that those with advanced liver disease should have normal standard of care (73) (Figure 2).

In conclusion, the risk of COVID-19 infection and its impact on chronic liver disease in children is still not entirely clear, although results from an Italian multicenter study suggest that a chronic liver condition is not an additional risk factor for severe disease course of COVID-19 in children.

### Auto-Immune Liver Disease and COVID-19

From the data documented above, children with chronic liver disease do not have an increased risk of infection with SARS-CoV-2 and have a milder disease course with little or no liver dysfunction compared to their adult counterparts (7, 68). In this section, we explore whether patients on immunosuppression for autoimmune liver disease (AILD) are more susceptible to COVID-19. Data from the published literature suggests adult patients with autoimmune liver disease (AILD) are not more susceptible to COVID-19 (7, 79, 80).

Di Giorgio et al. surveyed 148 AILD patients, of whom 47 were children and none tested positive for COVID-19 (37 with autoimmune hepatitis (AIH) and 11 with sclerosing cholangitis/overlap syndrome); four adult females tested positive, all with AIH on immunosuppression (IS). Of these, one elderly patient with comorbidities of hypertension and dyslipidaemia died from COVID pneumonia (7). Another survey from Bergamo found 5/138 (3.6%) AILD patients tested COVID-19+ve; all were female, four had AIH and one with PBC, on IS and all recovered fully without needing hospitalization (80).

In view of the low risk, EASL-ESCMID advises against immunosuppressive (IS) reduction in AILD, especially in low risk stable patients on longstanding IS. The empiric reduction or withdrawal of IS may inadvertently spark a disease flare and worsen disease activity and progression (73). Treatment with high dose steroids and subsequent IS dose escalation then places the patient at increased risk of infection along with their inherent side effects (80). Careful consideration on a case by case basis should be undertaken when considering lowering of immunosuppression in AILD patients with COVID-19 e.g., lymphopenia from purine synthase inhibition (73). This advice mirrors that given to liver transplant (LT) recipients (73).

Data from Lombardy and other centers suggests patients with AILD and/or inflammatory bowel disease (IBD) and transplanted patients are not at increased risk of COVID-19 (7, 80, 81).

The unifying factor in these patients, is that they are on IS. It appears that patients on IS who become infected with SARS-CoV-2 generally experience a milder disease (82). The apparent protective role of IS was also observed in the 2002 SARS-CoV1 epidemic, a virus which has an 80% genetic homology to SARS-CoV-2 and in the MERS epidemic in 2012 (83, 84). IS may be protective against COVID-19 by reducing the inflammation and severity of lung injury caused by the cytokine storm, and help modulate the immune host response against the virus (82, 84, 85).

None the less, it is important that AILD patients who need urgent care must be identified early and are not diverted from normal healthcare resources due to the pandemic (68, 73, 83). Particular attention should be paid to AIH/overlap syndrome patients with recurrent/severe disease flares, those with a history of cholangitis in PSC/overlap syndrome, decompensated liver disease and patients with severe portal hypertension who are at risk of GI bleeding (68, 73, 84).

It is important to be mindful that deranged liver function could be due to COVID and not the underlying AILD. This is especially true for stable patients with new onset liver dysfunction. A transient rise in transaminases as a result of general inflammation and circulating cytokines causing a “bystander hepatitis” is well-described in the literature (73, 84).

In addition, the majority of antivirals are metabolized by the liver (84). As discussed above, there is a potential for drug induced liver injury in patients on COVID-19 treatment. There is also a risk of drug interactions in patients on antivirals and IS; particularly for patients on Tacrolimus, the systemic level of which is vulnerable to acute changes in P450 cytochrome enzyme inhibitor activity as seen with the protease inhibitors (68, 86).

### COVID-19 in Pediatric Liver Transplant Recipients

Solid organ transplant recipients are in special attention during the SARS-CoV-2 pandemic. The associated life-long immunosuppression and possible comorbidities suggest an elevated risk for a severe course of COVID-19. Evidence in other viral respiratory infections, e.g., with influenza H1N1 (87), enhanced the presumed risk of a severe course of COVID-19. This section discusses the epidemiology, the clinical course, and the treatment of COVID-19 infections in adult and pediatric liver transplant (LT) recipients. Moreover, we search for evidence of risk factors determining a severe course of Sars-CoV-2 infection.

In children, only single case reports have been published on children with COVID-19 post-LT (Table 3) (8, 9). The outcome was favorable in all documented cases. The risk of symptomatic infection is increased in the immediate post-transplant course.

**Table 3:** Children with COVID-19 post-liver transplantation.

**Patient**	**Time post-LT**	**Clinical presentation**	**References**
55 months old, female	Onset 5 months post-LT	• Tachypnea, otherwise remarkable, moderately elevated AST/ALT • Coinfection with EBV • No specific treatment.	(8)
6 months old, female	Onset 4 days post-LDLT	• Fever, tachypnea POD 4 • Hydroxychloroquine • Graft hepatitis, LBx POD 7: mixed inflammatory infiltrate, interpreted as ACR → Banff score 5	(9)

A survey by the ERN Transplant-Child asked 18 European centers on the prevalence of COVID-19 infections in pLT recipients: 10/18 centers reported <10 patients with COVID-19 infection. Further, 89% of centers reported, that <5% of pLT recipients needed ICU treatment (10). A Single-center survey at a pediatric LT center in an area with high Sars-CoV-2 prevalence did not report any confirmed COVID-19 cases, although two children with close contact to a COVID-19 infected had mild respiratory symptoms, but infection with SARS-CoV-2 was not confirmed (11).

There have been several observational and register studies on SARS-CoV-2 infections in adult LT recipients. In general, adult LT recipients showed comparable courses of COVID-19 to non-transplant patients (88, 89). However, the severity and course of COVID-19 infections are mainly determined by extrahepatic comorbidities. Risk factors for mortality in adult LT recipients include age > 65 years, overweight, arterial hypertension and diabetes (88, 90). The outcome can be impaired by secondary bacterial or fungal infections (91). An international European study on COVID-19 in adult LT recipients documented a case fatality rate for the overall study population was 12% (95% CI 5 to 24%), whereas that of the hospitalized patients was 17% (95% CI 7 to 32%) (92).

Adult LT recipients with previous cancer had a worse outcome of COVID-19 infection. Interestingly, there was no difference between the fatality rate of hospitalized transplant recipients and the general population, which ranges from 15 to 22%. A Spanish registry, which included 111 infected adult LT recipients, noted that although the incidence of COVID-19 infections was higher in transplanted patients, they had a lower mortality rate of 18%, than the matched general population [standard-mortality-ratio = 95.5 (95%CI 94.2-96.8)], suggesting that they were protected by their IS therapy as discussed above (93). However, independent of the clinical course, the nasopharyngeal swab test and RNA PCR stays positive longer in immunosuppressed patients suggesting viral persistence and continued infectivity (94).

The immunosuppressive treatment regime may have a major impact on the course and outcome of LT recipients with COVID-19 (95). Early data recommended a reduction or even a withdrawal of IS on hospital admission for COVID-19 (12) but more recent studies show a more sophisticated picture: A case study from Northern Italy (96) and a US American registry study (90) found no correlation between IS and disease severity in adult and pediatric LT recipients during the Sars-CoV-2 epidemic. While moderate Tacrolimus levels were not associated with worse outcome, mycophenolate containing IS regime was an independent risk factor of severe COVID-19 (RR 3.94; p=0.003) (93). Moreover, pre-clinical and clinical evidence suggested that the early inflammatory response to the virus (e.g., IL-6 blood concentration) could be attenuated in immunosuppressed patients (97). Currently, international societies, including AASLD and EASL, do not recommend a reduction of immunosuppressive medication in liver transplant patients (73, 96). The risk of acute rejection and graft loss is increased and does not justify a withdrawal. Therefore, individual consideration of immunosuppressive therapy is necessary. While mycophenolate should be stopped, CNI based IS regime should be continued.

Several studies are evaluating specific antiviral treatment for COVID-19. Currently, only the early application of Remdesivir has proven a therapeutic effect in Sars-CoV-2 infections (64). Other drugs, including Lopinavir/Ritonavir are still under evaluation. In transplanted patients, drug interactions, especially with CNIs, including Tacrolimus, is likely (https://www.covid19-druginteractions.org/) and toxic Tacrolimus levels have been described with e.g., Lopinavir/Ritonavir (98). Despite this, antiviral treatment should be part of the management of severe COVID-19 infections (73) along with antibiotics (e.g., azithromycin) and antimycotic treatment in order to prevent secondary infections (99).

Further, anticoagulation with UFH or LMWH is recommended according to EASL/AASLD guidelines.

Transplantation of organs, particularly thoracic organs may transmit Sars-CoV-2 and so organs from Sars-CoV-2 positive donors are not accepted (73, 96) despite preliminary data that the risk of virus transmission is low in liver or heart grafts. Although organs from Sars-CoV-2 positive patients could be considered for selected patients with high waiting list mortality (100), further evidence would be required before implementation.

Implementation of preventive measures is essential and effective to prevent infection in LT patients (12, 101–103). These include social distancing, face masks and application of routine swab tests of patients in the pre- and post-transplant setting. Further, the implementation of telemedicine into the outpatient management of transplant patients can help to reduce patient contacts and the risk of infection (104).

Altogether the incidence of COVID-19 in pLT recipients is low, and there has been no evidence that immunosuppressed pLT recipients are under a higher risk for severe COVID-19 infections. The documented clinical courses in liver transplanted children were benign and mortality in both adults and children was very low (105). This early data can help to reassure patients and families, but further epidemiological studies are needed. Ultimately, the introduction of Sars-CoV-2 vaccination can help to reduce the risk of infections in immunosuppressed children.

## Summary and Conclusions

We are still learning about the short and long-term effects of COVID-19. Emerging data have documented different patterns of infection and disease outcome between adults and children, perhaps related to the difference in their innate and adaptive immunity. The initial impression that children with or without chronic disease are less likely to have severe or symptomatic disease appears confirmed in many reports worldwide.

Many adult studies have documented that patients with cirrhosis and or liver cancer are at a higher risk of infection with Covid-19 and are more likely to have a more severe course if they have the same associated comorbidities as other adults, but this has not been universally agreed.

Data on children with all forms of liver disease, whether auto-immune or post-transplant are scarce, although preliminary data of low rates of infection and morbidity and mortality are reassuring. The pathogenetic mechanisms of liver damage in either adults or children are not fully understood and the impact of COVID-19, or indeed the new variants of Covid-19, recently described, on the clinical outcomes in patients with liver disease is undefined.

Furthermore, there are no systematic data on infectivity rates in children with liver disease, the presence of asymptomatic carriage of the virus in this population nor systematic data on the long term outcome of these coronavirus infections in this group of immunocompromised children. Further research is needed to define liver involvement and the impact of COVID-19 in children with liver disease.

Based on current knowledge, much more needs to be clarified about the impact Covid-19 in this population of children with liver disease and/or post-transplant. As vaccination program begin, further consideration would need to be given to immune status and the response required to ensure adequate vaccination in this vulnerable population.

## Author Contributions

AD, SW, and SH: substantial contributions to the conception or design of the work, drafting the work, and final approval of the version to be published. DK: substantial contributions to the conception or design of the work, final drafting and revising it critically for important intellectual content, and final approval of the version to be published. All authors contributed to the article and approved the submitted version.

## Conflict of Interest

The authors declare that the research was conducted in the absence of any commercial or financial relationships that could be construed as a potential conflict of interest.
